# Bereaved Individuals Attempts to Explain Their Unexplainable Experiences Related to the Death of Their Loved one Through Attribution Thinking

**DOI:** 10.1177/00302228241277853

**Published:** 2024-08-24

**Authors:** Milla Mäkikomsi, Anja Terkamo-Moisio, Marja Kaunonen, Anna L. Aho

**Affiliations:** 1 Faculty of Social Sciences, 7840 Tampere University, Tampere, Finland; 2 Faculty of Medicine, 60655 University of Helsinki, Helsinki, Finland; 3 Faculty of Health Sciences, 205537 University of Eastern Finland, Kuopio, Finland; 4 648436 Wellbeing Services County of Pirkanmaa, Hospital Services, Tampere, Finland

**Keywords:** continuing bonds, unexplainable experiences, bereavement, meaning making, grief, Family, attribution thinking

## Abstract

Continuing bond manifesting as unexplainable experiences reflects bereaved´s attempts to restore connection with the deceased. As an experience unaligned with a person’s overall schemas of meaning unexplainable experiences are a source of anxiety that individuals aim to alleviate by attribution thinking. This study describes how bereaved aim to explain unexplainable experiences related to the death of their loved ones. The study analysed 408 narratives of 181 bereaved individuals. Bereaved individuals (1) described the meaning of their experience to be receaving information, emotions or support by the experience and communicating about crossing the boundary between life and death. As (2) reflections on the cause of their experiences bereaved individuals described certain and uncertain explanations of the phenomenon, ruling out explanations and the compatibility or incompatibility of the experience with their prior worldviews. The process of attribution thinking must be perceived as meaningful regardless of the outcome of the bereaved person’s reflection.

## Introduction

Continuing bond (CB) is an internalised representation of the bond between bereaved persons and their deceased loved ones that manifests in thoughts and actions in multiple ways (Field & Filanosky, 2009). CB may also manifest as unexplainable experiences (Jahn & Spencer-Thomas, 2014), including discussions with the deceased person, various sensory experiences or sensing the presence of the deceased person (Keen et al., 2013; Klass & Steffen, 2018). Unexplainable experiences may occur in the bereaved person’s physical, sensory or mental environment, and they are characterised by the bereaved person’s experience that these are connected to the death of their loved one (Mäkikomsi et al., 2021a). Rubin et al. (2009) have argued that women experience stronger continuing bonds (CBs), but drawing conclusions on the role of gender is hampered by the low number of male participants in CB research (Karydi, 2018).

CB also takes on different meanings for different bereaved individuals. If the relationship with the deceased individual has been good before the person’s death, it is more likely that the CB will continue to be perceived positively (Field et al., 2005; Karydi, 2018). Nevertheless, a negative or ambivalent relationship with the deceased person does not always result in negative continuing bond experiences; instead, the manifestations of CB may even serve as a means to fix the relationship between the deceased and the bereaved person (Root & Exline, 2014). The manifestations of CB emerging as unexplainable experiences may promote or impair the coping of the bereaved person (Mäkikomsi et al., 2021b). Factors such as the bereaved person’s attachment style contribute to whether the CB promotes coping (Tidwell et al., 2021). It is important to increase knowledge about post-death relationships manifesting as unexplainable experiences, as up to 10%–40% of people in Western countries have experienced CB with a deceased loved one (Beischel et al., 2015; Castro et al., 2014), despite these experiences being often culturally stigmatized in the Western world (Steffen & Coyle, 2011). It is due to this stigma that bereaved individuals avoid sharing their experiences and may therefore be excluded from the support necessary for processing the experiences (Chapple et al., 2011; Keen et al., 2013; Mäkikomsi et al., 2021b).

A religious or spiritual worldview and beliefs of the bereaved person are associated with a stronger CB or have been described to at least shape this bond and its significance to the bereaved person (Karydi, 2018). On the other hand, CB has been found to occur in various cultures and religious contexts as well as among people with different socioeconomic backgrounds (Beischel et al., 2015; Mäkikomsi et al., 2021a; Steffen & Coyle, 2011). However, the literature on the CB theory usually starts with an assumption that, for bereaved people, death signifies the end of the existence of the deceased person in one way or another (Root & Exline, 2014). According to Field et al. (2005), CB may either promote or impair the coping of the bereaved person depending on whether it is aligned with the person’s preconceptions and worldview. According to Karydi (2018), a stronger CB is linked to a more intense and prolonged experience of grief in adults who have lost their parents during their childhood. This may, however, be explicitly associated with the need of these adults for intense expressions of grief as a token of maintaining the connection with the deceased person and cannot necessarily be generalised to bereaved individuals as a whole.

Attachment security is widely perceived as one of the main paradigms for understanding the experiences and coping of bereaved individuals (Tidwell et al., 2021). An attachment relationship is a behavioural system grounded in biology that guides individuals to maintain or restore proximity to the object of their attachment (Bowlby, 1969, 1973, 1980). In early childhood, the most essential element of an attachment relationship involves the regulation of physical distance to the object of attachment. Later in life, establishing attachment security does not require physical proximity to the object; instead, understanding that this connection can be restored is enough. However, maintaining a sense of security requires at least the knowledge that physical proximity can be restored. (Ainsworth et al., 1978.) An inability to restore physical proximity often triggers a process in which the person refuses to believe what has happened and experiences separation anxiety which guides them to seek proximity with the lost object of affection (Bowlby, 1980).

Attachment has been shown to also play a key role in the CB phenomenon. The death of a loved one triggers the attachment system and the continuing bond may be perceived as an attempt to retain connection with the lost loved one. As a result, the key elements of CB include separation anxiety and a strong need to attempt to reconnect with the lost loved one. Such attempts may manifest as a need to visit places associated with the deceased person, spotting the deceased person in a crowd of people or hearing sounds related to the deceased person, for instance. Based on the attachment theory, the manifestations of CB can be considered as a fixed part of the process of coping with grief and coming to acceptance of the loss of a loved one. Indeed, the manifestations of CB should be perceived as meaningful links to the past for the bereaved person. However, manifestations of continuing bonds may also serve as internalized representations of the safety brought by the deceased person and they may be beneficial for coping with grief. Nevertheless, this requires that the bereaved person has accepted that their loss is permanent and that the manifestations do not cause anxiety to them. (Field et al., 2005; Tidwell et al., 2021.) The quality of the attachment relationship also affects the continuing bond. Bereaved individuals with several avoidant attachment characteristics are more likely to have weaker continuing bonds as they did not regard the object of affection as a major source of security when the person was still alive either (Currier et al., 2015; Tidwell et al., 2021).

Meaning-making is also closely connected to grief and the related process of coping. Finding meaning related to the death of a loved one mitigates severe complications in bereavement (Neimeyer et al., 2006). Mothers who have lost children to suicide have been found to alleviate the anxiety related to their loss by making sense of their loss through narration (Whalen & Tisdell, 2023). A meaning-making process carried out with a written disclosure method associating positive meanings to the bereavement experience may reduce grief reactions, depression and post-traumatic stress symptoms among bereaved individuals (Lichtenthal & Cruess, 2010). Instead of a process limited to individuals or those closest to them, the sense-making related to bereavement may also be perceived as a wider social process where individuals search for meaning in the deceased person’s life and death through social interactions (Neimeyer et al., 2014).

The process of finding purpose and explanations for CB manifestations may also be perceived as a part of adjusting to the loss of a loved one. According to the integrative model of meaning reconstruction in bereavement described by Gillies and Neimeyer (2006), Park (2010) and Tidwell et al. (2021), people aim to mitigate the anxiety associated with extremely stressful experiences through meaning reconstruction. General and situation-specific schemas of meaning lie at the core of the model. The general schemas of meaning are constructed in early development stages and provide a cognitive frame for processing experiences. Experiences that conflict with these schemas cause anxiety, which the individual seeks to mitigate by reducing the conflict. Attribution thinking which consists of making sense and finding significance in experiences plays a key role in this process. In this context, sense-making refers to finding an explanation for what caused the experience and how it affects the individual facing it. Meanwhile, significance refers to attributing value or purpose to the experience. Attribution thinking aims to shape the general or situation-specific schema of meaning to reduce the conflict between these two. The process includes conscious and unconscious, cognitive and emotional work that partly utilise the same mechanisms and are complementary to each other. While attribution thinking is a significant factor that mitigates anxiety especially when a person is faced with an event that they struggle to accept or creates a major conflict given his or her prior beliefs, its impact is strongly linked to the success of alleviating the conflict. (Gillies & Neimeyer, 2006; Park, 2010; Tidwell et al., 2021.) It is worth noting that CB manifestations that emerge as unexplainable experiences may, in themselves, cause conflicts in schema of meaning that occur simultaneously with the conflicts caused by the death of a loved one. As a result, it is important to gain a better understanding of the phenomenon from the perspective of supporting bereaved individuals.

Studies on the manifestations of continuing bonds and meaning-making are largely focused on examining the connection between the manifestations of CB and the process of coping with the death of a loved one. However, few studies have concerned the meaning-making related to the actual CB manifestations. According to Neimeyer et al. (2006), a strong CB with a deceased person is only associated with more severe separation anxiety if the bereaved person fails to find meaning to their loss. Chan et al. (2005) point out that Chinese bereaved persons consider the manifestations of CB to occur based on the initiative of the deceased or bereaved person, and their interpretations are characterised by the bereaved person’s preconceptions of death and life after death. In Chinese culture and conception of death, the manifestations of continuing bonds that emerge as experiences of reconnecting with a deceased person are perceived as normal and therefore a natural part of adjusting to loss. CB may also affect bereaved individuals’ experiences of significance related to work and through these, guide their choices (Hoppes & Segal, 2010). A study concerning older people who had lost their spouse found that, for bereaved participants for whom the connection with their spouse had been a major factor contributing to meaning in life, a strong CB continued to support the experience of significance after the spouse’s death (Vähäkangas et al., 2022).

The purpose of this study is to describe how bereaved persons who have lost a loved one attempt to find sense and meaning in the manifestations of CB emerging as unexplainable experiences. The aim is to increase understanding of how bereaved persons aim to find an explanation for the unexplainable experiences they have had in connection with the death of their loved one and through these promote opportunities for bereaved individuals to receive better support for processing their unexplainable experiences. Increasing understanding also helps normalise the unexplainable experiences related to the death of a loved one and the related process of meaning-making to reduce the stigma associated with the experiences.

## Methods

The qualitative research method was selected for this study as the aim was to describe the phenomenon as authentically, diversely and extensively as possible. The starting point for this study was the interpretivist paradigm, based on which the way bereaved people interpret their experiences plays a focal role and interpretations are always considered to emerge in relation to the person’s prior experiences (Yanow, 2013). Although research seeks to provide a description that is as authentic as possible, it is also important to acknowledge that each researcher will interpret the experiences through their respective frame during the research process (McChesney & Aldridge, 2019). Interpretation must nonetheless be perceived as a key research tool that aims to describe the meanings attached by a bereaved person to their experiences. The phenomenological hermeneutical approach is applied, involving understanding the experiences described by the bereaved persons as real, meaningful experiences that can be verbalised. (Yanow, 2013).

### Recruitment of Participants and Data Collection

The research participants were recruited to the study with an open research announcement using websites and social media platforms aimed at bereaved users. The data were collected in the period 2013–2020 with an online form that asked bereaved respondents to describe their unexplainable experiences related to the death of a loved one. The question did not determine separate domains for the descriptions. The respondents were asked for their background information, which included age, self-rated health, employment or student status, level of education, relationship status, religious conviction and the time passed since the death of the loved one. The background information collected on the deceased person included gender, age at death, relation to the bereaved person, cause of death and whether or not the death was expected. The research participants were bereaved individuals (*n* = 181) whose loved ones had died regardless of the cause of death of the deceased person, the relationship between the deceased and the bereaved person, or the time passed since the death of the loved one. In their narratives, the bereaved persons described their unexplainable experiences related to death at or after the time of death of the deceased loved one. The study analysed the narratives (*n* = 408) concerning unexplainable experiences related to the death of a loved one.

### Ethical Aspects

Ethical issues were considered consistently throughout the research prosess. The ethical pre-assessment from the Research Ethics Committee in Finland was not required to this study according to current Finnish legislation and research guidelines (Tutkimuseettinen neuvottelukunta, 2019). The research participants gave their consent for using their narratives in research after receiving written information about the aim and purpose of the study, the processing of their data, and the reporting and utilisation of the dataset. The data were processed confidentially and no individual participants can be identified from the research report. The research process was carried out in compliance with integrity and meticulousness. The researcher in charge of data analysis did not have personal experiences or strong preconceptions of the studied phenomenon and the research group had no interests that may have influenced the results of this study. The research group had strong competence in the used methods and research related to the topic. (Tutkimuseettinen neuvottelukunta, 2019). To support the quality of the research article, the Standards for Reporting Qualitative Research (SRQR) checklist prepared by the EQUATOR network was applied (O’Brien et al., 2014).

### Data Analysis

In the examination of the frequency and percentage distributions of the background variables utilised the SPSS version 27 software. The qualitative data were analysed by one researcher using inductive content analysis with the help of the Atlas-ti 8 software. The other researchers commented on the analysis at its different phases. Narrative excerpts that answered the research question were identified from the data and subsequently reduced. Reduced expressions with shared content were combined and the analysis was continued to subcategories and upper categories which were named according to their content. The data were structured and abstracted during the analysis process but retaining authentic information was ensured by revisiting the data and original expressions several times during the process. At the final analysis phase, the data had been saturated to the point that no more new information emerged at this point. (Bengtsson, 2016).

## Results

### Description of Participants

All the research participants were women (Table 1). The participants’ average age was 44 years, with the youngest participant being 16 and the oldest 69 years old. The majority of the participants rated their health as very good or good by their own assessment (70.2 %). Most of the participants had completed at least upper secondary education (61.9 %) and the majority of them were either employed or studying (70.9 %). More than half of the participants were in a relationship (56.9 %) and the majority were members of some religious community (81.3 %). For most of the participants, less than five years had passed since the death of their loved one (72.4 %) and most had more than one unexplainable experience (71.0 %).

**Table 1.** Characteristics of the Study Participants.

Variable	*N*	Valid %
Age		
16–20 years	4	2.4
21–30 years	22	12.9
31–40 years	38	22.3
41–50 years	57	33.6
51–60 years	36	21.2
61–70 years	13	7.6
Self-evaluated health status		
Very good	21	11.6
Quite good	106	58.6
Mediocre/Satisfactory	44	24.3
Quite or very poor/bad	10	5.6
Working life participation		
Full-time employment	91	51.2
Part-time employment	13	7.3
Unemployed/furloughed	9	5.1
Retired	14	7.9
On sick leave	10	5.6
Stay-at-home parent/caregiver	19	10.7
Student	22	12.4
Education level		
Basic education	9	5.0
Vocational training	18	9.9
Vocational diploma	42	23.2
Secondary level vocational degree	54	29.8
University of applied sciences degree	28	15.5
University degree	30	16.6
Marital status		
Married	74	40.9
In a domestic partnership	29	16.0
Single	23	12.7
Divorced/separated	22	12.2
Widowed	33	18.2
Religious denomination		
Evangelical lutheran	143	78.6
Other	5	2.7
None	33	18.1
Time since the death of the loved one		
Less than a year	40	22.1
1–5 years	91	50.3
6–10 years	26	14.4
More than 10 years	24	13.3

The majority of the deceased persons were male (64.1 %) (Table 2). Most (60.5 %) had been over 18 years old at the time of death, with the average age at death being 32 years. The deceased person was most commonly the bereaved person’s child or grandchild (56.4 %) but the data also contained narratives of bereaved respondents who had lost their spouses or ex-spouses (24.3 %), siblings or friends (11.6 %) and parents or grandparents (7.7 %). The most common cause of death was an illness (44.0 %). Other causes of death in the narratives contained by the dataset included suicide (20.9 %), accidents (18.7 %) and stillbirth or cot death (13.2 %). The death had been unexpected for more than half of the participants (59.7 %).

**Table 2.** Background Variables of the Deceased Loved One.

Background variable	*N*	Valid. %
Gender of the deceased person		
Male	116	64.1
Female	65	35.9
Age of death		
Foetal stage	25	13.1
Infancy (less than 1 year)	11	7.2
Childhood (1–12 years)	22	12.4
Adolescence (13–17 years)	12	6.8
Early adulthood (18–29 years)	40	22.6
Adulthood (30–49 years)	38	21.5
Late adulthood (50–69 years)	24	13.6
Old age (more than 70 years)	5	2.8
The role of the deceased person in the participant’s life		
Child or grandchild	102	56.4
Spouse or ex-spouse	44	24.3
Sibling or close friend	21	11.6
Parent or grandparent	14	7.7
Cause of death		
Illness	80	44.0
Accident	34	18.7
Stillbirth/Cot death	24	13.2
Suicide	38	20.9
Homicide	4	2.2
Unknown at the time of participation	2	1.1
Forewarning of death		
No forewarning	108	59.7
Forewarning	73	40.3

### Attribution Thinking Related to Unexplainable Experiences of Bereaved Individuals

The bereaved respondents applied attribution thinking to find an explanation for their experiences, looking for meaning and cause for them. By looking for meaning in the experiences, the bereaved persons aimed to find an answer to the question of why the experience happened. In their attempts to find the cause of the experience, the bereaved persons aimed to understand what caused the phenomenon they experienced.

#### Perceived Meaning of Unexplainable Experiences

The bereaved participants felt that the meaning of their experiences had been to convey information or emotions, receive support and communicate about crossing the boundary between life and death. Conveying information included passing on information, receiving instructions, and warning of dangers. The bereaved individuals felt they received information about the death of their loved one, the identity of the person causing the death as well as answers to questions they had posed through experiences. The bereaved persons also felt their deceased loved one had instructed them in managing their issues and contacting other loved ones. Based on the bereaved respondents’ explanations, the deceased persons had alerted them in traffic or warned them that a person they had met was harmful.

“2 weeks after their death, they appeared in my home and came to thank me for arranging for their children to meet up.

“I was sitting on the terrace and looking at the side of my car’s front bumper that my daughter had once scratched. I was thinking: “That’s where you, [name of deceased person] left an indelible mark...” At the same time, the blinkers of the car flashed twice, giving me the answer “y-es”.

“I’ve also heard bumps or creaks on the partition door (folding door) separating my bedroom from my living room as warning signals if I’ve tried to get involved with “bad men”.

The bereaved persons felt that their deceased loved ones were conveying emotions to express their delight and distress. According to the bereaved persons’ experiences, the deceased loved ones wished to express their delight, such as doing well, being free from suffering, gratitude and accepting the choices made by the bereaved persons. The bereaved respondents also felt that the deceased persons were expressing their distress, including yearning, regretting the events leading to their death, anger for their death, displeasure with others touching their belongings and sadness related to spending money.

“I felt as if my son had come over to tell me “Mother, I’m doing alright now.”

“The second time it was like they tried to get me to leave with them because they were missing me so much.”

“I sensed that he was angry because people were touching his belongings. My father was very particular about his things.”

The respondents considered their unexplainable experiences to have the meaning of receiving support when the deceased person was interpreted as wanting to help the bereaved person or improve their mood. The deceased person was considered to help the bereaved person cope with a difficult situation and solve problems, mitigate the pain felt by the bereaved person, cure their illness, support their intimate partnership and provide protection against threats. The bereaved persons also felt that their deceased loved ones aimed to improve their mood by permitting them to feel joy, alleviating their guilt, bringing comfort, creating faith in the future, cheering them up and calming them down and joking around with them.

“That’s when we knew our little girl wanted to help us stay together and bear with it all!”

“I felt that the deceased person wanted to comfort me.”

The respondents also felt that, through unexplainable experiences, the deceased person aimed to inform them about having crossed the boundary between life and death. The deceased loved ones were considered to remind the bereaved person of their existence and express a continuing connection with the bereaved person, and their wish to let go of this world or visit it. The bereaved persons felt that their deceased loved ones wanted to express their existence by giving a sign, greeting and reminding the bereaved person of their existence and flaunting their temperament. The respondents felt that the deceased persons wished to express the continuing bond with the bereaved person by giving them faith in reuniting with them and communicating about continued involvement in their life. The deceased persons were considered to express their desire to bid farewell to the world by saying goodbye and instructing the bereaved person to let go of them. The experiences were also considered to signify the deceased person’s desire to visit this world by following what the bereaved person is doing or visiting their home.

“That’s when I laugh out loud and say. Well, aren’t you in a bad mood, throwing objects around like that. And I always get this feeling that [the deceased person] is saying. No, I’m not, it’s just that that’s the only way for me to get your attention.”

“I immediately just felt that it was my sister visiting me to greet me and remind me that she’s still present in our lives.”

“I’m certain that my little brother came to say goodbye to me.”

“They just stood there quietly and I could immediately sense that they just wanted to be there in silence and watch me get ready for work.”

#### Perceived Causes of Unexplainable Experiences

Unexplainable experiences related to the death of a loved one stirred a need to reflect on the cause of the phenomenon in the bereaved persons. The bereaved individuals’ reflections on the cause of their experiences were divided into certain and uncertain explanations of the phenomenon, ruling out explanations and the compatibility or incompatibility of the experience with their prior worldviews. The bereaved individuals felt certain that the phenomenon they had experienced had been caused by death, angels, a godlike figure, their own mind or the deceased person. Uncertain explanations for the phenomenon reflected by the bereaved individuals included disturbed mental health, imagination, coincidence, situational factors, tactile memory, sleep and supernatural aspects. The bereaved persons also ruled out experiences based on explanations such as the effect of medications, psychoses, coincidence, regular dreams or sleeping, possible situational factors and a failure to reproduce the phenomenon.

“I knew that my spouse came to visit me to tell me he/she is always with us.”

“Whether or not that’s my imagination, God only knows.”

“I even tried to recreate the situation by creating various air phenomena but I couldn’t reproduce it, not even by blowing a candle from one side.”

Regarding the compatibility of the unexplainable experience with their prior worldview, the bereaved persons described their previous unexplainable experiences related to the deceased loved one or the death of another loved one as well as a previous strong belief in spirituality or a general inclination to having unexplainable experiences. Meanwhile, examples of the incompatibility of the unexplainable experience with the bereaved person’s prior worldview included a previously sceptical view of supernatural explanations, a lack of religious conviction and the unique nature of the unexplainable experience in the person’s life.

“I’ve never experienced any other supernatural phenomena.”

“I experienced the same phenomenon when my father died.””I’ve believed in spirituality and the spirit world for a long time, it’s something that has helped me move forward.”

## Discussion

This study indicated that bereaved individuals have versatile interpretations of the meaning of their unexplainable experiences and that these were strongly linked to the communications between the bereaved person and their deceased loved one. This is a relevant observation, as previous research has shown that the manifestations of CB emerging as unexplainable experiences typically contain communicative elements (Keen et al., 2013; Klass & Steffen, 2018, Mäkikomsi et al., 2021b). Bereaved individuals use attribution related to unexplainable experiences to look for meaning in the experience itself as well as the death of their loved one. It is significant that in all the narratives, the meaning of the experience was perceived as positive or neutral and none of the bereaved participants reported a negative or threatening meaning for their experience. Field et al. (2005) and Tidwell et al. (2021) have emphasised the key role of the attachment theory in understanding the experiences of bereaved persons. Similarly, in the present study, the deceased loved ones can be considered to have represented security to the bereaved person even to the extent that they were considered to still aim to positively influence the bereaved person’s life after death. This observation is in line with the view presented by Field et al. (2005) based on which the manifestations of CB may serve as internalised representations of security for the bereaved person. Indeed, strengthening the element of security may play a key role when supporting bereaved persons after the loss of a loved one.

On the other hand, this study revealed that bereaved individuals feel that the deceased person aims to explain the factors related to their death, providing at times highly concrete information about the conditions leading to their death, which can be considered to reflect the need of the bereaved person to find an explanation to the death of their loved one. This finding is aligned with the integrative model of meaning reconstruction in bereavement (Gillies & Neimeyer, 2006; Park, 2010; Tidwell et al., 2021.), according to which the most important purpose of the attempts of meaning-making is to mitigate anxiety. It appears that the meaning associated with unexplainable experiences partly serves the need to find an explanation for the experience itself and partly the need to find an explanation for the death of a loved one. The meaning attributed to some of the experiences was letting go, which can be considered significant to accepting the finality of the death, which was also found to support the usefulness of the experiences in coping with grief in a study by Field et al. (2005). Future research should explore whether the meaning-making process by bereaved individuals could be supported in a way that helps them adapt their thinking towards a direction that supports their coping.

In this study, the bereaved persons mirrored their unexplainable experiences to their conceptions of unexplainable experiences, religiosity and spirituality before the incidence. Also according to Karydi (2018), religious and spiritual beliefs adjust CB and its significance to the bereaved person. However, in our study, those reporting manifestations of CB emerging as unexplainable experiences included both bereaved persons with similar previous experiences as well as those who had never experienced anything like it or who did not even believe in the existence of paranormal or spiritual phenomena. In fact, we may argue that the phenomenon of unexplainable experiences related to the death of a loved one is distinct from an inclination to unexplainable experiences in other contexts. It may be necessary to further clarify this in future studies to better separate the phenomenon from hallucinations or experiences related to religious activities, for instance.

From the perspective of bereaved individuals, it is important to encounter the experiences in the correct framework to provide the persons with the right kind of support in processing their experiences. In selecting the type of support provided, it may be relevant to assess whether the bereaved person’s experiences match their prior worldview; by the integrative model of meaning reconstruction in bereavement, the conflict caused by the incompatibility of the experience and the person’s prior worldview is what causes anxiety to the bereaved person, and this can be mitigated by adjusting either the general or situation-specific frame of interpretation (Gillies & Neimeyer, 2006; Park, 2010; Tidwell et al., 2021.) Therefore, the support necessary for processing the experience should probably target the success of this consolidation process regardless of the person’s previous worldview related to unexplainable experiences. It is also important to note that stigmatising an experience that fits the bereaved person’s world of experiences as a hallucination, for example, may impair the person’s coping by creating a conflict between an external interpretation and the bereaved person’s own meaning-making system.

The attribution thinking related to unexplainable experiences of bereaved individuals contains elements of both rational and emotional explanations and their combinations, which reflects the view of meaning-making as a cognitive-emotional process that involves assessing the experience in relation to a previously constructed cognitive frame of interpretation (Gillies & Neimeyer, 2006; Park, 2010; Tidwell et al., 2021.) As some of the bereaved participants included reflections on alternative explanations in their narratives, explaining one’s experience may also be perceived as a part of the meaning-making process. Bereaved individuals also seek explanations for their experiences both by evaluating the workings of their mind as well as external circumstances. It is illustrative of the unique nature of the experiences that some of the bereaved participants had attempted to recreate their experience by influencing controllable external factors. Bereaved people also aim to rationalise their experiences by ruling out alternative explanations. Indeed, it is important to make room for any kind of reflection to allow the bereaved person to make progress with their internal process and allow them to reach a conclusion that they find satisfying.

### Limitations

All the research participants were women. This can be considered a clear limitation and renders the results not generalizable to the experiences of all bereaved individuals. Nevertheless, this may support the results of previous studies based on which such experiences are more common among women (Rubin et al., 2009). However, when making interpretations, the high prevalence of studies that only include female participants in the literature on continuing bonds should be taken into consideration (Karydi, 2018). It is also possible that women are generally more active than men in participating in studies. Indeed, it would be important to target separate studies on the experiences of CB among bereaved men in the future to obtain knowledge about the possible gendered nature of the experiences.

## Conclusion

This study described how bereaved individuals aim to explain their unexplainable experiences related to the death of a loved one by reflecting on their meaning and cause. An attachment bond between the bereaved and deceased person manifesting through unexplainable experiences may provide the former with a sense of security. On the other hand, the meaning-making process related to explaining the experiences also serves as an adaptation tool for the bereaved person, and promoting this process may support the coping of bereaved individuals. This study significantly increases the understanding of manifestations of CB that emerge as unexplainable experiences, as little previous research is available on the related meaning-making and attribution.

The professionals encountering bereaved individuals must understand that manifestations of CB that emerge as unexplainable experiences are meaningful for bereaved persons and they can be considered to have a purpose even if the phenomenon cannot be explained. Based on this study, the meaning of the experiences was as important to the bereaved individuals as explaining the phenomenon, as their narratives included simultaneous descriptions of both. As many of the bereaved participants also reflected on various alternatives as the explanation for their experience in their narratives without coming to a conclusion, this reflection itself can be considered to be important to bereaved individuals. The significance of finding meaning and an explanation for the experiences is also emphasised by the fact that the participants were not separately requested to describe these elements, but they instead spontaneously produced these as a part of their personal experience narratives. From the perspective of supporting bereaved individuals, it is essential to understand that according to the integrative model of meaning reconstruction, the conflict between the experience and the person’s previous worldview is what causes anxiety in the bereaved individuals. As a result, support measures must also be planned with an understanding of the bereaved person’s worldview.

## References

[bibr1-00302228241277853] Ainsworth M. D. S. Blehar M. C. Waters E. Wall S. (1978). Patterns of attachment: A psychological study of the strange situation. Erlbaum.

[bibr2-00302228241277853] Beischel J. Mosher C. Boccuzzi M. (2015). The possible effects on bereavement of assisted after-death communication during readings with psychic mediums: A continuing bonds perspective. Omega: The Journal of Death and Dying, 70(2), 169–194. 10.2190/OM.70.2.b 25628023

[bibr3-00302228241277853] Bengtsson M. (2016). How to plan and perform a qualitative study using content analysis. NursingPlus Open, 2, 8–14. 10.1016/j.npls.2016.01.001

[bibr4-00302228241277853] Bowlby J. (1969). Attachment and loss. In: Attachment (1). Basic Books.

[bibr5-00302228241277853] Bowlby J. (1973). Attachment and loss: Vol. 2 separation: Anxiety and anger. Basic Books.

[bibr6-00302228241277853] Bowlby J. (1980). Attachment and loss (3). Basic Books. *Loss: Sadness and depression* .

[bibr7-00302228241277853] Castro M. Burrows R. Wooffitt R. (2014). The paranormal is (still) normal: The sociological implications of a survey of paranormal experiences in Great Britain. Sociological Research Online, 19(3), 1–15. 10.5153/sro.3355

[bibr8-00302228241277853] Chan C. L. W. Chow A. Y. M. Ho S. M. Y. Tsui Y. K. Y. Tin A. F. Koo B. W. K. Koo E. W. K. (2005). The experience of Chinese bereaved persons: A preliminary study of meaning making and continuing bonds. Death Studies, 29(10), 923–947. 10.1080/07481180500299287 16265798

[bibr9-00302228241277853] Chapple A. Swift C. Ziebland S. (2011). The role of spirituality and religion for those bereaved due to a traumatic death. Mortality, 16(1), 1–19. 10.1080/13576275.2011.535998

[bibr10-00302228241277853] Currier J. M. Irish J. E. Neimeyer R. A. Foster J. D. (2015). Attachment, continuing bonds, and complicated grief following violent loss: Testing a moderated model. Death Studies, 39(4), 201–210. 10.1080/07481187.2014.975869 25551174

[bibr11-00302228241277853] Field N. P. Filanosky C. (2009). Continuing bonds, risk factors for complicated grief, and adjustment to bereavement. Death Studies, 34(1), 1–29. 10.1080/07481180903372269 24479173

[bibr12-00302228241277853] Field N. P. Gao B. Paderna L. (2005). Continuing bonds in bereavement: An attachment theory perspective. Death Studies, 29(4), 277–299. 10.1080/07481180590923689 15849880

[bibr13-00302228241277853] Gillies J. Neimeyer R. A. (2006). Loss, grief, and the search for significance: Toward a model of meaning reconstruction in bereavement. Journal of Constructivist Psychology, 19(1), 31–65. 10.1080/10720530500311182

[bibr14-00302228241277853] Hoppes S. Segal R. (2010). Reconstructing meaning through occupation after the death of a family member: Accommodation, assimilation, and continuing bonds. American Journal of Occupational Therapy, 64(1), 133–141. 10.5014/ajot.64.1.133 20131573

[bibr15-00302228241277853] Jahn D. R. Spencer-Thomas S. (2014). Continuing bonds through after-death spiritual experiences in individuals bereaved by suicide. Journal of Spirituality in Mental Health, 16(4), 311–324. 10.1080/19349637.2015.957612

[bibr16-00302228241277853] Karydi E. (2018). Childhood bereavement: The role of the surviving parent and the continuing bond with the deceased. Death Studies, 42(7), 415–425. 28816620 10.1080/07481187.2017.1363829

[bibr17-00302228241277853] Keen C. Murray C. Payne S. (2013). Sensing the presence of the deceased: A narrative review. Mental Health, Religion & Culture, 16(4), 384–402. 10.1080/13674676.2012.678987

[bibr18-00302228241277853] Klass D. Steffen E. (2018). Introduction: Continuing bonds - 20 years on. In Klass D. Steffen E. (Eds.), Continuing bonds in bereavement, new directions for research and practice (pp. 38–61). Routledge.

[bibr19-00302228241277853] Lichtenthal W. G. Cruess D. G. (2010). Effects of directed written disclosure on grief and distress symptoms among bereaved individuals. Death Studies, 34(6), 475–499. 10.1080/07481187.2010.483332 24482856 PMC3909885

[bibr20-00302228241277853] Mäkikomsi M. Terkamo-Moisio A. Kaunonen M. Aho A. L. (2021a). Unexplained experiences in the context of bereavement – qualitative analysis. Mortality, 28(3), 443–459. 10.1080/13576275.2021.1991903 PMC10768326 34866475

[bibr21-00302228241277853] Mäkikomsi M. Terkamo-Moisio A. Kaunonen M. Aho A. L. (2021b). Consequences of unexplained experiences in the context of bereavement – qualitative analysis. Omega: The Journal of Death and Dying, 88(3), 936–950. 10.1177/00302228211053474 34866475 PMC10768326

[bibr22-00302228241277853] McChesney K. Aldridge J. (2019). Weaving an interpretivist stance throughout mixed methods research. International Journal of Research and Method in Education, 42(3), 225–238. 10.1080/1743727X.2019.1590811

[bibr23-00302228241277853] Neimeyer R. A. Baldwin S. A. Gillies J. (2006). Continuing bonds and reconstructing meaning: Mitigating complications in bereavement. Death Studies, 30(8), 715–738. 10.1080/07481180600848322 16972369

[bibr24-00302228241277853] Neimeyer R. A. Klass D. Dennis M. R. (2014). A social constructionist account of grief: Loss and the narration of meaning. Death Studies, 38(8), 485–498. 10.1080/07481187.2014.913454 24738824

[bibr34-00302228241277853] O’Brien B. C. Harris I. B. Beckman T. J. Reed D. A. Cook D. A. (2014). Standards for Reporting Qualitative Research, A Synthesis of Recommendations. Academic Medicine, 89(9), 1245–1251. DOI:10.1097/ACM.0000000000000388 24979285

[bibr25-00302228241277853] Park C. L. (2010). Making sense of the meaning literature : An integrative review of meaning making and its effects on adjustment to stressful life events. Psychological Bulletin, 136(2), 257–301. 10.1037/a0018301 20192563

[bibr26-00302228241277853] Root B. Exline J. (2014). The role of continuing bonds in coping with grief: Overview and future directions. Death Studies, 38(1), 1–8. 10.1080/07481187.2012.712608 24521040

[bibr27-00302228241277853] Rubin S. S. Bar Nadav O. Malkinson R. Koren D. Goffer-Shnarch M. Michaeli E. (2009). The two-track model of bereavement questionnaire (TTBQ): Development and validation of a relational measure. Death Studies, 33(4), 305–333. 10.1080/07481180802705668 19368062

[bibr28-00302228241277853] Steffen E. Coyle A. (2011). Sense of presence experiences and meaning making in bereavement: A qualitative analysis. Death Studies, 35(7), 579–609. 10.1080/07481187.2011.584758 24501839

[bibr29-00302228241277853] Tidwell B. L. Larson E. D. Bentley J. A. (2021). Attachment security and continuing bonds: The mediating role of meaning-made in bereavement. Journal of Loss and Trauma, 26(2), 116–133. 10.1080/15325024.2020.1753389

[bibr30-00302228241277853] Tutkimuseettinen neuvottelukunta . (2019). Ihmiseen kohdistuvan tutkimuksen eettiset periaatteet ja ihmistieteiden eettinen ennakkoarviointi Suomessa. Tutkimuseettisen neuvottelukunnan ohje 2019. In Guidelines of the research ethical committee 2019]. Tutkimuseettisen neuvottelukunnan julkaisuja 3/2019. [The Ethical Principals of Studies Concerning Human Subjects and Ethical Pre-assessment of Human Sciences in Finland. https://tenk.fi/sites/default/files/2021-01/Ihmistieteiden_eettisen_ennakkoarvioinnin_ohje_2020.pdf

[bibr31-00302228241277853] Vähäkangas A. Saarelainen S.-M. Ojalammi J. (2022). The search for meaning in life through continuing and/or transforming the bond to a deceased spouse in late life. Pastoral Psychology, 71(1), 43–59. 10.1007/s11089-021-00979-w

[bibr32-00302228241277853] Whalen G. C. Tisdell E. J. (2023). Black and blue butterflies: The transformative journeys of mothers who lost a child to suicide. Journal of Transformative Education, 21(2), 207–224. 10.1177/15413446221085463

[bibr33-00302228241277853] Yanow D. (2013). Thinking interpretively: Philosophical presuppositions and the human sciences. In Yanow D. Schwartz-Shea P. (Eds.), Interpretation and method: Empirical research methods and the interpretive turn (pp. 5–26). Taylor & Frances Group. https://ebookcentral.proquest.com/lib/tampere/detail.action?docID=302458

